# Prof. O. W. Holmes’ Microscope

**Published:** 1867-02

**Authors:** 


*Prof. O. W. Holmes' Microscope.*—A meeting of the Essex Institute, Salem, Mass., was held Tuesday, May 1, 1866, to bring together all the miscroscopes which could be conveniently obtained, for the purpose of interesting the friends of the Institute in this department of science; also to celebrate, in an appropriate manner, the ancient festival of the first of May.

The meeting proved a decided success, over three hundred members and their friends being present, besides a number of invited guests, among whom were, Prof. O. W. Holmes, of Boston; Dr. A. A. Gould, Vice-President of the Boston Society of Natural History, and others.

Prof. Holmes, on being introduced, briefly pointed out the distinction between the simple and the compound miscroscope, and describing the method by which the imperfections of the latter have been remedied within the last forty years, he proceeded to speak of the more remarkable improvements it has received at the hands of American opticians and philosophers.

1. Enlargement of the angle of aperture. In 1852, Mr. Quekett said in his well known-treatise, quoting Mr. Andrew Ross, the most famous of London makers of microscopes, that 135° was the largest angular pencil which could be passed through a microscopic object glass. But long before this an American optician had made a $\frac{1}{12}$ object glass having an angular aperture of 146°, the same glass which he now held in his hand. Since that time the same maker has made glasses with an angular aperture thirty degrees and more larger than this. Mr. Webb will show you in connection with his beautiful binocular a glass having an angle of 178°, which, as he says, and as we should expect, is equal to the resolution of the most difficult tests.

The audacious American, who carried the angle of aperture more than forty degrees beyond the limits of the possible (according to the highest English authority), was Mr. Charles Spencer, of Canastota, a small town in the midst of half-burned stumps of the forest in the interior of the State of New York.

2. Next on the list of American inventions and improvements, comes the inverted microscope of Dr. J. Lawrence Smith, of Louisiana, a form of instrument universally approved and very widely adopted by chemists as particularly fitted for their investigations.

3. The binocular microscope of Professor Riddle, of New Orleans, which, variously improved and modified, is now extensively employed both in England and on the continent, as well as in this country.

The best known American miscroscope makers are, Mr. Spencer, the pioneer among them, whose inventive genius has stimulated the opticians of the Old World to attempt feats which they considered impossible until he showed they could be and had been done; Mr. Tolles, his worthy successor, whose glasses challenge competition with any in the world; Mr. Wales, not so long known among us, but making first-rate objectives; Mr. Grünow, whose instruments of moderate cost are perhaps the best the American student can buy, and who can make excellent microscopes of costlier pattern when required; Mr. Zentmayer, whose stands are equal, if not superior, in elegance and workmanship to the finest of European make.

Dr. Holmes then said, that, at the risk of taxing the powers of belief of those before him, he would attempt to give some idea of what is meant by an enlargement of twenty thousand diameters, within a fraction of which these objects are amplified.

It means that their surface, or that portion of it which you see, looks four hundred million times as large as it really is.

If your thumb nail were thus magnified, it would cover 18 acres of ground.

A fly, weighing one grain, thus magnified in surface and in like proportion in thickness, so as to keep his proper figure, if his weight increased at the same rate as his bulk, would weigh as much as a million horses rolled into one great monster horse.

A man weighs a million grains, and magnified in each dimension, as much as these dots are enlarged, would weigh as much as a billion horses; more very probably than ever lived on this planet, from the Adam of horseflesh to the present time.

Many who are here this evening remember the famous moonhoax of Richard Adams Locke. It seems not impossible that the fancy of enlarging telescopic images by the misroscope so as to bring out details upon the surface of the satellite may yet be realized. If the moon looks about a foot in diameter, it would if enlarged as these dots are enlarged, be extended to nearly four miles in diameter, say, if you will, $\frac{1}{600}$ of its actual diameter. This would give us pictures of everything on the scale of one inch to fifty feet, and would show us men and women, if such there were on the moon's surface, as exceedingly interesting little animals of about the size of certain insects held in small esteem by the human species, and very easily seen as well as felt.

Errors excepted, of course, in the above calculations, which are believed, however to be essentially correct.

Dr. Holmes then said that he had been particularly requested to bring with him the microscope which he is in the habit of using, the mechanical arrangements of which are of his own contrivance. The glasses shown in connection with it were, a $\frac{1}{2}$, a $\frac{1}{4}$, and a $\frac{1}{12}$, made by Spencer.

The principal points in the simple and inexpensive arrangement to which he called attention were the following:—

1. Fixed wooden stand, carrying with it lamp for direct illumination, objectives, eye-glasses, and other apparatus.

2. Tube supported on two forks cut in the wood; inclined at an angle of 35°; rotated by turning the shade-disk, which is 8 inches in diameter, and thus regulating the focal distance by the movement of a brass check which bears against an inclined surface of glass, giving a rapid and medium adjustment.

3. Delicate secondary adjustment, by a screw with scalloped head, placed close to the thumb which with the forefinger moves the object stage. This screw depresses very slightly one of two brass plates, fixed to object stage, against which the glass object slide is pressed forward by two small springs. This arrangement has the incidental advantages of bringing all objects to the same level, and of affording protection to the thin glass of the side.

4. Horseshoe magnet for fixed stage.

5. Object stage of soft iron, 8 inches long, 1 inch and $\frac{1}{4}$ wide, adhering to fixed stage by attraction, assisted by brass spring at one end, loaded to keep it down, moves horizontally by sliding over an edge $\frac{3}{4}$ of an inch to the left of middle of fixed stage, and up and down in the arc of a circle of which this edge is the centre. Requires but one hand for management, which hand is always in position to command the fine adjustment.

6. Achromatic condenser (or any other piece of apparatus) slides in between the branches of the horseshoe magnet. The diaphragm is directly behind the achromatic condenser.

7. A small plano-convex lens occupies one hole of the diaphragm, and is very useful in concentrating the rays of oblique light when that is used.

8. Although this instrument is arranged chiefly with reference to using the direct rays of a lamp without any reflection, a mirror can be substituted for the lamp if desired. The following is Dr. Holmes' method of arranging this adjunct:—A plano-convex lens is set in a frame, to be used as a condenser. Two plane mirrors of the same size are cemented back to back. This double mirror fits against the plane side of the lens, thus giving a plane mirror on one side and the equivalent of a concave mirror on the other.

9. Dr. Holmes employs a simple *indicator*, made by sticking a portion of a fine needle to the diaphragm of the eye-glass with a bit of wax. This is a great convenience in demonstrations, it being easy to bring any particular object of examination to the point of the needle by moving the stage.

10. A very convenient complement of the instrument here shown is the simple arrangement shown by Quekett (fig. 257, 2d edition), which is especially adapted for very low powers, for dissecting, examining the circulation, etc.,

Dr. Holmes said he would take occasion to mention a plan he had lately adopted for preserving recent preparation of soft tissues so that they could be shown day after day. It is simply laying them on a wet cloth, which is itself placed on a sheet of India-rubber cloth, and covering them with a bell-glass. The air being soon saturated with moisture, the preparations cannot dry.

He then exhibited one of Mr. Lockhart Clarke's sections of the spinal cord, and a single nerve-cell isolated and stained with carmine, prepared by Gerlach, both of which were lent him by Dr. Dean, to whom they were presented by the distinguished anatomists of whose skill they are singularly perfect specimens.—*Dental Cosmos.*

